# Discovery of Viviparous Fish in Louisiana

**Published:** 1855-01

**Authors:** 


### ECLECTIC DEPARTMENT,

### AND SPIRIT OF THE MEDICAL PERIODICAL PRESS.

### *Discovery of Viviparous Fish in Louisiana.*

NEW ORLEANS, Nov. 1, 1854.

"What have we here? A fish—a strange fish."—*The Tempest.*

In the month of October, 1854, through the politeness of J. C. B. Harvey, M. D., of Tchoupitoulas street, I received a small osseous fish, caught in the New Orleans canal, which connects the city with Lake Pontchartrain. This fish had been placed in a basket containing crabs, one of which wounded it slightly in the abdomen near the cloaca, thereby exposing several fœtal fish, enveloped in a delicate membrane. The parent fish, which had been rudely thrust into a narrow mouthed vial of spirits, retains after immersion for two weeks, the original *rigor mortis*, and the same remark applies to the fœtuses, though they have been soaked in water; some of them have been forcibly straightened. On the 17th of October, in the presence of, and assisted by, Drs. J. Hale and M. Dowler, I enlarged the wound and proceeded to dissect a somewhat globular mass of fœtuses bounded by the intestines before, and separated from them by an indescribably thin, diaphanous membrane; this mass was further bounded above by the spine and ribs, below and behind by the posterior inferior abdominal walls, bulging backward of the anal orifice and fin. The exterior envelope of this oblong globe consisted of a very thin, pelucid, extremely delicate and apparently laminated and flocculent membrane, like the amnion of the human embryo in the early state; it did not form a simple sack, but consisted of many duplications like the arachnoidal reflections among the sinuosities and convolutions of the human brain, sending its prolongations as the hyaloid membrane does, through the vitreous mass of the eye.

This uterine membrane (ovisac it may not be termed) contained twenty-two fishes. It is probable that the inner surface of the uterine membrane sent forth a still more delicate membrane which enveloped each fish after the manner that the peritoneum envelopes the abdominal viscera; but the parent fish, and still more its inclosed organs, were to admit of full demonstration during a necessarily hurried examination; moreover, the wish not to mutilate the parent fish very much prevented a fuller dissection of the fœtal mass *in situ.*

Each fœtal fish was doubled laterally, sometimes to the right, sometimes to the left, into the globular form, the caudal fin which is inclined to the lancet shape, though blunter, overlapped one eye and one side of the mouth; each fish *in situ*, and even after forcible extraction from its bed, was infolded in a sack; some were drawn out united by pedicles to a common stem, somewhat like an umbilical cord.

These fœtal fishes presented a perfect example of close packing. A perceptible force was required to dislodge them from their beds. The concavity left by their extraction appeared to be lined with a smooth, black, peritoneal membrane.

The intestines, which were very minute, were crowded forward by the rounded mass of fœtuses which occupied the greater portion of the abdominal cavity. No ova were discovered.

The maternal fish not being much mutilated, is reserved for a more detailed technical description, which my leisure and the limits of this journal will not admit of at present.

Without attempting fully to describe even the dermal skeleton, I may observe that this tiny fish is a most symmetrical one. Its minuteness may be imagined when I state that after the removal of the inclosed fœtuses it weighed only seven grains, though not disemboweled. Thorough desiccation would probably reduce its weight about half or more. The fish exposed for two hours in the shade on a damp day, was but slightly desiccated. It was weighed by Mr. Macpherson, apothecary, in my presence; but fearing a mistake, I had it weighed again, with the same result. If each fœtus should weigh but one grain, the aggregate would be more than three times greater than that of the mother!

Measurement in inches: Length, including the caudal fin, 2 inches; greatest circumference, $1\frac{3}{4}$; width, vertically, $\frac{1}{2}$; length of thoracic fin, $\frac{1}{4}$; the caudal fin does not expand from its base or proximal end, but terminates ovally, its length $\frac{1}{2}$; the anal but little expanded, $\frac{1}{4}$; the ventral is too minute for convenient measurement, being almost invisible without a lens; the dorsal, which is single, has but a slight vertical width, arising from a base $\frac{1}{4}$ of an inch, nearly opposite, though a little forward of the anal.

The teeth are advanced, nearly ranging with the lips, being very numerous, close and small, though scarcely discernible without a magnifying glass. Lips thin, the under one slightly projecting; angles of the mouth not depressed; eyes medium size; head flattened at the frontal bone; operculum much expanded. The branchiæ largely developed in three great arches, densely fringed with thick tufts, the outer and inner rows inclining to the central, having also, one, perhaps more rows behind, which are shorter.

The predominant hue of this fish is a tawny or fawn color; the opercula silvery; head metallic gray; muzzle blackish, slightly projecting.

There are six rows of rather quadrangular black spots, more particularly marked in the posterior half of the body, averaging twenty-five spots for each row. These black spots, resting on a tawny ground, leaving intervals something larger than themselves, give a picturesque appearance, forming stripes of alternating hues, the three upper of which slightly curve, corresponding to the arching back; but each becomes straighter, the fourth and fifth being nearly straight; the sixth or lower row follows the abdominal curve, and disappears at the anal fin; the other five rows gradually converge without coalescing at the origin of the caudal fin. At the orign of this fin the spots are displaced out of line. By this arrangement the six rows of alternating black and tawny leave in the longitudinal direction six other tawny stripes, all of which, except the two interrupted ones, lost at the anal fin, converge without mingling in the tail, all being about equal in length. The colors fade somewhat into a greenish yellow around the thoracic fins, which are nearly central between the dorsum and abdomen, being on a level with the eyes, and about one line from the opercula.

There are six or seven rows of scales. The spinous rays of the fins are about twenty-five caudal, twelve anal, fifteen dorsal, ten thoracic.

These fœtuses are half an inch long, all alike, exactly resembling the maternal form and proportion, with the following slight exceptions, namely: their bodies are more slender and compressed laterally; their heads are comparatively larger, and their eyes more prominent; their colors are less variegated, and paler; a still greater difference appears about the middle of the abdomen, where there is attached to each fœtus a whitish, faintly yellowish placental-like irregularly formed mass of considerable size, having a broad base, being apparently implanted in or blended with the abdominal integument, possessing considerable strength, and constituting what may be termed the umbilical prominence; perhaps, it may turn out upon further examination that this mass may be not placental, but an adherent mesenteric mass of convoluted membrane.

These fœtal fishes were probably sufficiently developed at the time of the parent's death to live independent of the mother.

It appears from the proceedings of the Academy of Natural Sciences of Philadelphia, for 1854, that Dr. Gibbons, of the Academy of Natural Sciences of San Francisco, "claims priority of description of viviparous fish," in behalf of the gold-shimmering waters of California, and consequently that State takes precedence over Louisiana. Agassiz, whose sounding (fishing) line has passed the living waters to the most ancient palæozoic rocks, says, in regard to the California viviparous fishes, that a "country which furnishes such novelties in our days, bids fair to enrich science with many other unexpected facts." B. DOWLER, M. D.

—*N. O. Med. and Surg. Journal.*

